# Sheep-to-Human Transmission of Orf Virus during Eid al-Adha Religious Practices, France

**DOI:** 10.3201/eid1901.120421

**Published:** 2013-01

**Authors:** Antoine Nougairede, Christelle Fossati, Nicolas Salez, Stephan Cohen-Bacrie, Laetitia Ninove, Fabrice Michel, Samer Aboukais, Mathias Buttner, Christine Zandotti, Xavier de Lamballerie, Remi N. Charrel

**Affiliations:** Author affiliations: Aix-Marseille University, Marseille, France (A. Nougairede, N. Salez, L. Ninove, X. de Lamballerie, R.N. Charrel);; Assistance Publique–Hopitaux de Marseille, Marseille (A. Nougairede, L. Ninove, C. Zandotti, X. de Lamballerie, R.N. Charrel);; Hôpital Paul Desbief, Marseille (C. Fossati);; AlphaBio Laboratory, Marseille (S. Cohen-Bacrie);; Service Santé, Protection Animales et Environnement, Marseille (F. Michel);; Regional Office of the French Institute for Public Health Surveillance (Institut de Veille Sanitaire), Marseille (S. Aboukais);; Bavarian Health and Food Safety Authority, Oberschleißheim, Germany (M. Buttner)

**Keywords:** parapoxvirus, Orf virus, cutaneous infection, transmission, religious practices, Eid al-Adha, Eid al-Kabir, diagnosis, zoonoses, sheep, viruses, *Parapoxvirus* genus, family *Poxviridae*

## Abstract

Five persons in France were infected with Orf virus after skin wounds were exposed to infected sheep tissues during Eid al-Adha, the Muslim Feast of Sacrifice. Infections were confirmed by electron microscopy, PCR, and sequence analysis. Prevention and control of this underdiagnosed disease can be achieved by educating physicians, slaughterhouse workers, and persons participating in Eid al-Adha.

Orf virus (genus *Parapoxvirus*, family *Poxviridae*) is endemic to most countries. The virus primarily causes contagious ecthyma in wild and domestic ruminants, mostly sheep and goats (*1*). Human infections caused by occupational and household exposures have been described (*2*–*5*); they most commonly cause lesions on the hands (*1*,*3*,*6*). We report Orf virus infection in 5 humans who had household exposure to the virus.

## The Cases

**Figure 1:**
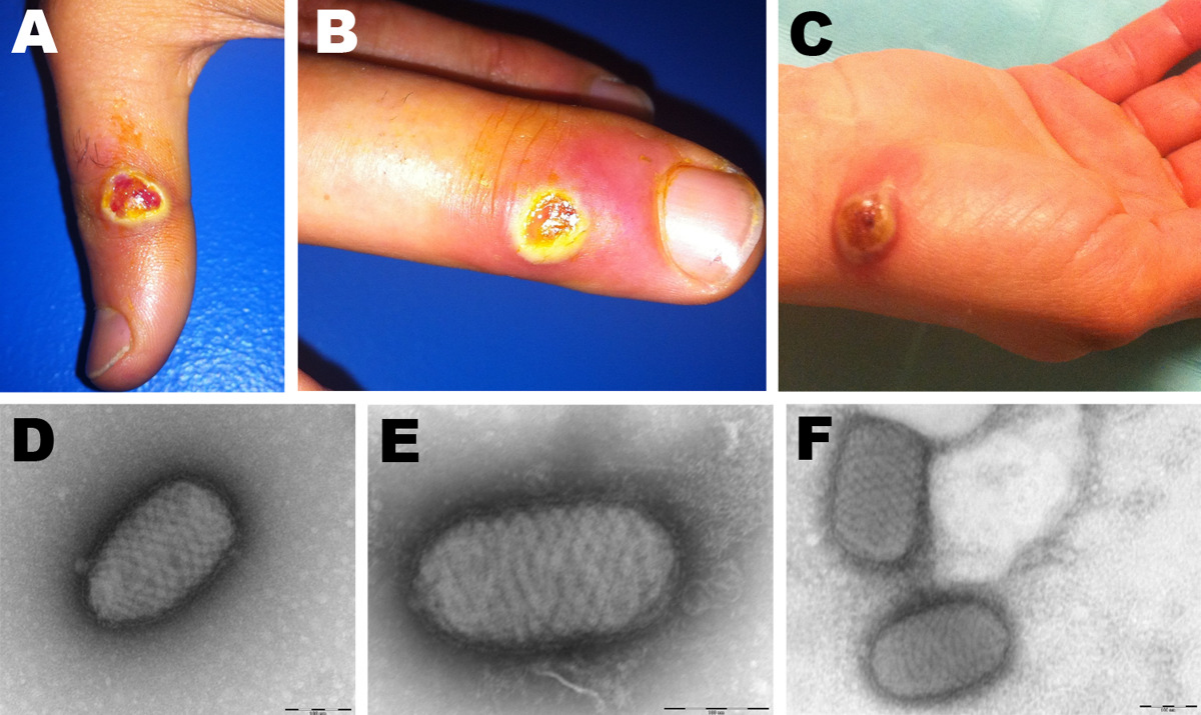
Orf virus infection in 5 persons who butchered or prepared lambs as part of a religious practice for Eid al-Adha (the Muslim Feast of Sacrifice), Marseille, France, 2011. Cutaneous lesions on hands of case-patient 3 (A, B) and case-patient 5 (C) are shown. Negative-staining electron microscopy of samples from case-patient 3 (D) and case-patient 5 (E, F) show ovoid particles (≈250 nm long, 150 nm wide) with a crisscross appearance; the size and appearance of these particles are highly suggestive of parapoxvirus virions.

Case-patient 1, a 51-year-old woman, was examined on November 28, 2011, by a surgeon (CF) for an 8-mm lesion without local complications on her left thumb. Case-patient 2, the 33-year-old niece of case-patient 1, was hospitalized on November 29 for surgical excision of a phlegmonous lesion on the fifth finger of her left hand; she also had a fever and a lesion without local complications on the second finger of the same hand. The surgery was performed by CF, the same surgeon who examined case-patient 1. By chance, case-patient 3, the 38-year-old brother of case-patient 2, met CF on November 29 while visiting his sister in the hospital; he asked CF if she would examine lesions on his left hand. Clinical examination revealed 2 lesions (1 each on the thumb and third finger) without local complications (Figure 1, panels A and B). The man reported having fever and malaise 1 day before the examination.

While examining case-patient 3, CF became aware that case-patients 1–3 were members of the same family and that they had butchered or handled several lambs on November 6, 2011, in preparation for Eid al-Adha (also called Eid al-Kabir), the Muslim Feast of Sacrifice. Using a smartphone, CF photographed the lesions on case-patient 3 and transmitted the photographs to 2 infectious disease specialists. The specialists indicated that the lesions appeared to be typical of parapoxvirus infection. CF interviewed case-patients 1–3 again, and they reported having knife wounds after preparing lambs for the religious feast and seeing lesions on the gums and tongue of 1 lamb. Swab (Virocult; Medical Wire and Equipment Co. Ltd., Corsham, United Kingdom) specimens were obtained from lesions on case-patients 1 and 3 and sent, along with a surgical skin biopsy specimen from case-patient 2, to the virology laboratory at Public Assistance–Hospitals of Marseille, Marseille, France.

Case-patient 4, a 64-year-old woman, sought medical care on December 9 for an ulcerovegetative lesion on the third finger of her left hand; the lesion was on the internal face of the interphalangeal joint, and phlegmon and cellulitis were present. The patient reported that she had injured herself with a kitchen knife on November 6 while butchering lamb meat for Eid al-Adha. Ten days later, she noticed vesicular lesions at the injury site; a pustule complicated by superinfection subsequently developed. The lesion was surgically excised on December 12, and skin biopsy samples were sent to the virology laboratory at Public Assistance–Hospitals of Marseille.

Case-patient 5, a 42-year-old woman, sought medical care on December 14 for a painful 2-cm papulonodular lesion on her right wrist (Figure 1, panel C). She recalled being injured on November 6 with a knife used to cut off the head of a lamb that was being prepared for Eid al-Adha. The lesion was surgically excised, and a sample was sent to the virology laboratory at Public Assistance–Hospitals of Marseille.

**Figure 2:**
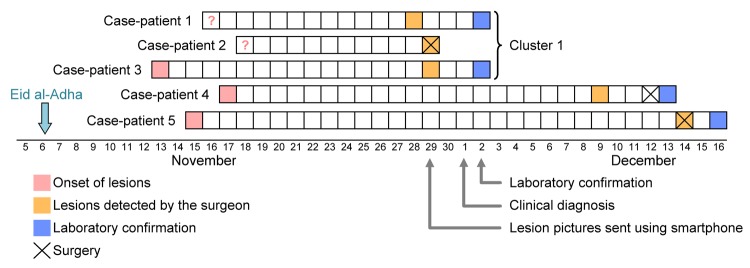
Natural history of Orf virus infection and diagnosis in 5 persons who butchered or prepared lambs as part of a religious practice for Eid al-Adha (the Muslim Feast of Sacrifice), Marseille, France, 2011. Arrows indicate events for the first cluster of cases among 3 related persons (a brother and sister and their aunt).

Specimens from case-patients 1–3 were received at the laboratory on December 2. Negative-stain electron microscopy (EM) was immediately performed: images revealed typical poxvirus-like particles in the specimen from case-patient 3 (Figure 1, panel D). To detect the presence of poxviruses, we subjected the samples to 2 broad-range PCRs with high-GC and low-GC primers (*7*). A 627-bp product was amplified with the high-GC primers from samples from case-patients 1 and 3 (Technical Appendix Figure 1). The products were directly sequenced, and results were subjected to BLAST analysis, which confirmed Orf virus infection (*8*). Skin-biopsy specimens from case-patients 4 and 5 were received at the laboratory on December 13 and 15, respectively, and processed as described above. EM revealed pox-like particles in both samples (Figure 1, panel E–F), and both were positive for Orf virus by PCR (Technical Appendix Figure 1). Figure 2 shows the time from lesion onset to laboratory diagnosis of Orf virus infection for case-patients 1–5.

Immediately after Orf virus infection was confirmed, we attempted virus isolation by cell culture, using Vero cells (the only cells available at the time) in 12.5-cm^2^ flasks; none of the samples yielded infectious virus. We later attempted virus isolation again, using fetal bovine esophagus cells, and isolated Orf viruses from samples from case-patients 3 and 5.

The 4 partial sequences obtained from samples from case-patients 1 and 3–5 were identical (GenBank accession no. JQ596637). We used ClustalX (*9*) to align the sequences for comparison with other homologous Orf virus sequences and other high-GC poxviruses. We performed phylogenetic analysis by using the neighbor-joining method (jukes-cantor algorithm) in MEGA5.0 software (*10*) (Technical Appendix Figure 2).

County veterinary services traced the origins of the sheep considered to be responsible for these human cases of Orf virus infection. The first 3 cases were linked to an illegal slaughterhouse within the county where sheep from France and Spain had been housed for 1 month. Case 4 was linked to sheep carcasses that were purchased from 2 legally operating butchers. Case 5 was linked to a certified temporary slaughterhouse that had sheep from regional counties, Spain, and Romania.

## Conclusions

Although human Orf virus infections have typically been associated with occupational animal contact (*1*–*3*,*6*), they also have been linked to Muslim religious practices and, more globally, to household meat processing or animal slaughter (*4*,*11*–*15*). Our findings show that clinical microbiology laboratories (other than national reference centers) can accurately detect and identify poxviruses by using EM and broad-spectrum PCR, such as that described by Li et al. (*7*). Our results also suggest that PCR is highly sensitive for detection of poxviruses, and they show that samples obtained by Virocult swab are well-suited for detection of Orf virus by EM or PCR and could replace more invasive methods (e.g., skin biopsy).

The finest, unblemished animals (e.g., cows, goats, sheep) were initially reserved for the ritual sacrifice during Eid al-Adha. Today, however, Muslims in developed countries (especially in cities) mostly buy lambs, which are cheaper and more plentiful but also highly susceptible to Orf virus infections (*1*). This change in buying practices has created a large market for possibly infected animals and an associated potential health risk for persons who butcher and prepare the animals.

The cases reported here stress the need for using appropriate measures to prevent animal-to-human transmission of pathogens. Public health officials should educate persons with occupational or household exposure to animals about the possibility for disease transmission and ways to avoid infection. Persons at increased risk for exposure to Orf virus include livestock owners, slaughterhouse workers, and persons who prepare animals at home for religious practices. Persons who handle animals should wear nonpermeable gloves, avoid exposure of open wounds, and meticulously wash skin wounds with soap and water after handling animals (*4*). In addition, slaughterhouses should verify that all animals to be sold or butchered are in good health; animals with Orf virus lesions should be disposed of in a safe manner. Physicians, including dermatologists, should be informed of the potential for Orf virus infection, a heretofore underdiagnosed disease (*15*), and suspected infections should be confirmed by microbiology laboratories. The first 3 cases presented here were rapidly diagnosed, and emergency department physicians were promptly advised of the cases, enabling rapid detection and confirmation of the subsequent cases.

Technical Appendix Phylogeny of Orf virus sequence of virus isolates and results of agarose gel electrophoresis of PCR products from virus isolates from Orf virus–infected persons, Marseille, France.
