# Supplementary material for: Sheep-to-Human Transmission of Orf Virus during Eid al-Adha Religious Practices, France
Source: Emerg Infect Dis. 2013 Jan;19(1):102–5. doi: 10.3201/eid1901.120421 (PMC3557981; doi:10.3201/eid1901.120421)
Supplement: Technical Appendix — Phylogeny of Orf virus sequence of virus isolates and results of agarose gel electrophoresis of PCR products from virus isolates from Orf virus–infected persons, Marseille, France. [file 12-0421-Techapp-s1.pdf]

Article DOI: http://dx.doi.org/10.3201/eid1901.120421

Sheep-to-Human Transmission of Orf Virus during Eid al-Adha Religious Practices, France

Technical Appendix

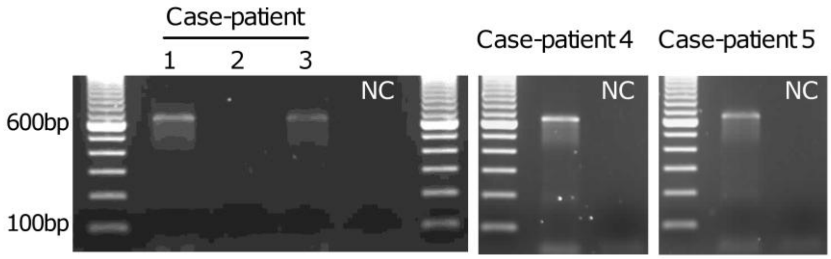

Technical Appendix Figure 1. Agarose gel (1.5%) electrophoresis of 627-bp PCR products from samples from persons exposed to Orf virus while butchering or preparing lambs as part of a religious practice for Eid al-Adha (the Muslim Feast of Sacrifice), Marseille, France, 2011. PCR products were obtained by using high-GC primers. NC, negative control.

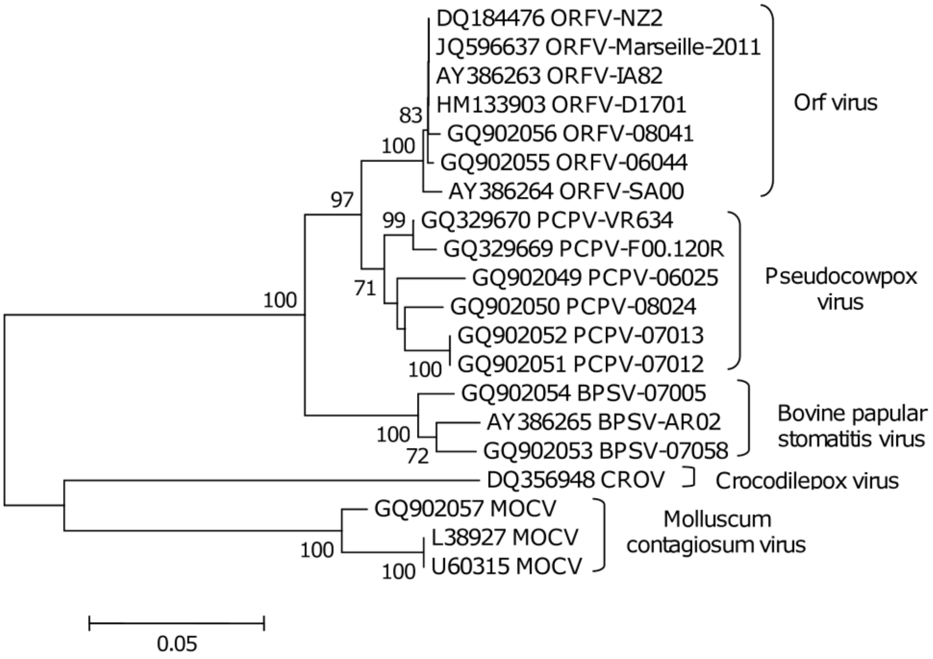

Technical Appendix Figure 2. Phylogeny of Orf virus isolated from 5 persons who butchered/prepared lambs as part of a religious practice for Eid al-Adha (the Muslim Feast of Sacrifice), Marseille, France, 2011.

Sequences of the Orf virus isolates are compared with homologous sequences for other *Parapoxvirus* spp. viruses. The phylogenetic tree was based on nucleotide sequences in the RNA polymerase subunit gene; the tree was constructed by using the neighbor-joining method. Sequence information for each virus corresponds to GenBank accession number/acronym/strain. Bootstraps values >70% are indicated (1,000 replicates).
